# Global burden of anticancer drug-induced acute kidney injury and tubulointerstitial nephritis from 1967 to 2023

**DOI:** 10.1038/s41598-024-67020-x

**Published:** 2024-07-12

**Authors:** Soo-Young Yoon, Sooji Lee, Kyeongmin Lee, Jin Sug Kim, Hyeon Seok Hwang, Andreas Kronbichler, Louis Jacob, Ju-Young Shin, Jin A. Lee, Jaeyu Park, Hyeri Lee, Hayeon Lee, Kyunghwan Jeong, Dong Keon Yon

**Affiliations:** 1 Division of Nephrology, Department of Internal Medicine, Kyung Hee University Hospital, Kyung Hee University College of Medicine, Seoul, South Korea; 2 Department of Medicine, Kyung Hee University College of Medicine, Seoul, South Korea; 3 Center for Digital Health, Medical Science Research Institute, Kyung Hee University College of Medicine, Seoul, South Korea; 4 Department of Regulatory Science, Kyung Hee University, Seoul, South Korea; 5 Department of Internal Medicine IV, Nephrology and Hypertension, Medical University Innsbruck, Innsbruck, Austria; 6 Research and Development Unit, Parc Sanitari Sant Joan de Déu, CIBERSAM, ISCIII, Barcelona, Spain; 7 Department of Physical Medicine and Rehabilitation, Lariboisière-Fernand Widal Hospital, AP-HP, Université Paris Cité, Paris, France; 8 Epidemiology of Ageing and Neurodegenerative Diseases (EpiAgeing), Inserm U1153, Université Paris Cité, Paris, France; 9 School of Pharmacy, Sungkyunkwan University, Suwon, South Korea; 10 Department of Biomedical Engineering, Kyung Hee University, Yongin, Korea; 11 Department of Pediatrics, Kyung Hee University Medical Center, Kyung Hee University College of Medicine, 23 Kyungheedae-Ro, Dongdaemun-Gu, Seoul, 02447 Republic of Korea

**Keywords:** Anticancer drugs, Acute kidney injury, Tubulointerstitial nephritis, World Health Organization, Kidney, Cancer

## Abstract

This study aims to figure out the worldwide prevalence of anticancer therapy-associated acute kidney injury (AKI) and tubulointerstitial nephritis (TIN) and the relative risk of each cancer drug. We conducted an analysis of VigiBase, the World Health Organization pharmacovigilance database, 1967–2023 via disproportionate Bayesian reporting method. We further categorized the anticancer drugs into four groups: cytotoxic therapy, hormone therapy, immunotherapy, and targeted therapy. Reporting odds ratio (ROR) and information component (IC) compares observed and expected values to investigate the associations of each category of anticancer drugs with AKI and TIN. We identified 32,722 and 2056 reports (male, n = 17,829 and 1,293) of anticancer therapy-associated AKI and TIN, respectively, among 4,592,036 reports of all-drug caused AKI and TIN. There has been a significant increase in reports since 2010, primarily due to increased reports of targeted therapy and immunotherapy. Immunotherapy exhibited a significant association with both AKI (ROR: 8.92; IC_0.25_: 3.06) and TIN (21.74; 4.24), followed by cytotoxic therapy (7.14; 2.68), targeted therapy (5.83; 2.40), and hormone therapy (2.59; 1.24) for AKI, and by cytotoxic therapy (2.60; 1.21) and targeted therapy (1.54; 0.61) for TIN. AKI and TIN were more prevalent among individuals under 45 years of age, with a female preponderance for AKI and males for TIN. These events were reported in close temporal relationship after initiation of the respective drug (16.53 days for AKI and 27.97 days for TIN), and exhibited a high fatality rate, with 23.6% for AKI and 16.3% for TIN. These findings underscore that kidney-related adverse drug reactions are of prognostic significance and strategies to mitigate such side effects are required to optimize anticancer therapy.

## Introduction

The recent strides in cancer therapeutics have been remarkable, reflecting persistent endeavors in drug development across the medical domain, despite cancer still ranking as the second leading cause of death globally^1^. From 2000 to 2019, the global life expectancy increased by 6 years, a trend largely attributable to the substantial rise in the global incidence of cancer and the utilization of diverse anticancer agents for therapeutic interventions^2,3^ The introduction of novel targeted therapies, immunotherapies and hormone therapies over the past decades has enhanced patient survival rates compared to standard conventional chemotherapies for specific types of cancers^4^.

It is well established that cancer patients, especially those with advanced cancer, are more susceptible to acute kidney injury (AKI) and tubulointerstitial nephritis (TIN)^5,6^. AKI in cancer patients impacts prognosis and management, including reduced rates of cancer remission^7^ and subsequently elevated mortality rates^8^ as administration of effective therapies is withheld due to concerns about kidney injury. Although advanced cancer itself could be a risk factor for AKI, there is a possibility that anticancer treatment could be a potential inducer of AKI and TIN^5^.

In particular, the various side effects associated with traditional cytotoxic therapies such as nephrotoxicity, are well-documented, especially with agents like cisplatin^9^.

Nonetheless, substantial concerns remain due to the relatively limited accumulation of experience with these novel drugs, especially concerning the diverse patient population, which encompasses variations in age, gender, and ethnicity. As the mechanisms of action of each drug vary widely, the mechanisms causing side effects are also diverse. While some studies have attempted to elucidate the nephrotoxicity of novel drugs, research on the long-term and large-scale effects of various medications remains insufficient^10^.

Our study focuses on analyzing the global risk of AKI and TIN linked to 165 types of anticancer medications. To assess the association between anticancer treatment and AKI and TIN, we have compared the risk to that of other drugs. Utilizing data from the World Health Organization (WHO) global pharmacovigilance database, our objective is to enhance patient safety following anticancer treatment and to establish effective monitoring guidelines for healthcare professionals. Vigibase, the WHO global database of adverse drug reactions (ADRs) reports, is essential resource for pharmacovigilance research due to its extensive coverage. Through the provision of comprehensive data which was observed in patients, our study aims to aid healthcare professionals in selecting safer and more appropriate medications for cancer patients in clinical settings.

## Methods and materials

### Study design and data sources

This retrospective pharmacovigilance study conducted a disproportionality analysis based on ADRs reported in the WHO database, VigiBase. VigiBase has amassed adverse case reports from over 170 countries, covering more than 25,000 drugs and compiling reports since 1967. It serves as a database system where adverse events can be reported by physicians, pharmacists, healthcare professionals, and patients^11,12^. Each incoming report undergoes scrutiny based on predefined quality criteria, and is regularly reviewed and analyzed. The Uppsala Monitoring Centre (UMC) in Uppsala, Sweden, manages the database, and thoroughly reviewed according to predefined quality standards. Reported adverse reactions are classified according to the preferred terms of the Medical Dictionary for Regulatory Activities (MedDRA) 26.0. Anticancer drug-related cases were extracted from November 14, 1967, to July 26, 2023. This study received approval from the Institutional Review Board at Kyung Hee University Medical Center and the Uppsala Monitoring Centre (WHO Collaborating Centre) and involved the utilization of de-identified patient data. The requirement for informed consent was waived in this study, as VigiBase does not contain personal information.

### Definition of exposure groups

We compiled a list of anticancer drugs included in our research, which was analyzed based on the anticancer drugs reported to the WHO in relevant literature^13^. Anticancer drugs were classified into four categories: (1) cytotoxic therapies, (2) hormone therapies, (3) immunotherapies including programmed cell death protein 1 (PD-1) inhibitors, programmed cell death ligand 1 (PD-L1) inhibitors, and cytotoxic T-lymphocyte-associated antigen 4 (CTLA-4) inhibitors, and (4) targeted therapies including kinase inhibitors. We excluded certain drugs from our study, even though they fell into one of the four categories for classifying anticancer agents. For instance, drugs like cyclophosphamide have dual roles, serving as both therapeutic agents for conditions like glomerulonephritis that may cause AKI and as anticancer agents^14,15^. This dual usage complicates distinguishing whether AKI results from their therapeutic use or their role as anticancer agents. Additionally, drugs like interferon have various components, such as alpha and beta, introducing potential confusion^16,17^.

All anticancer drugs are considered ‘suspected’ only when showing disproportionate association with kidney-related ADRs based on the WHO causality assessment recommendations. Each case report includes patient characteristics (sex and age), general information (region and reporting year), anticancer drug details (indication, start and end dates of administration, and dosage), and kidney-related adverse event information (time-to-onset and end date, seriousness, and final outcome). Time to onset refers to the number of days from the date of drug administration to the date when the adverse reaction occurred. The outcome of each event was classified as either "fatal" or "recovered/recovering," and severity, including hospitalization, lasting disabilities, life-threatening situations, and death, was assessed by physicians.

### Definition of outcomes

The primary outcome was the disproportionate measures of kidney-related ADRs after prescription of each anticancer drug classes. We divided kidney-related ADRs into AKI and TIN. Secondary outcomes included subgroup analyses of kidney-related ADRs following different types of anticancer drugs based on age (0–17, 18–44, 45–64, and ≥ 65 years) and sex. Disproportionality analysis was also conducted for each individual anticancer drug.

### Statistical analysis

We assessed whether suspected cases of AKI and TIN, classified based on the four types of anticancer drugs mentioned earlier, were differentially reported when compared to the entire pharmacovigilance database drugs. Two common pharmacovigilance measures of disproportionate analysis, the reporting odds ratio (ROR) and information component (IC) were calculated based on anticancer drugs. We used the ROR as a measure of association to estimate the frequentist disproportionality association. ROR is a statistical measure derived from a contingency table based on the number of ADRs. It compares the probability of a specific event occurring with a particular drug to the probability of the same event occurring with all other drugs not related to that specific event^18^. A lower 95% confidence interval (CI) of the ROR ≥ 1 is deemed statistically significant, indicating an association between the drug and a particular ADR.

IC is calculated using Bayesian methods for case-non-case analysis, comparing the adverse event rate of a specific drug to that of all other drugs^18,19^. The formula for calculating the IC is as follows: IC = log_2_([N_observed_ + 0.5]/[N_expected_ + 0.5]). N_observed_ is the case reports for a specific adverse reaction with a drug, N_expected_ is the expected cases for that drug-effect combination, calculated as [N_drug_ x N_effect_]/N_total_. N_drug_ stands for the number of case reports involving a specific drug, N_effect_ signifies the number of case reports for a particular reaction, and N_total_ encompasses the overall count of case reports within the database. IC_0.25_ is the lower limit of IC's 95% CI and a positive IC_0.25_ indicates a statistical signal^20^. A more detailed description is described in the Supplementary Methods. Disproportionality analysis and subgroup analysis of reports from health professionals were conducted to validate our results. All analyses were performed using the SAS version 9.4 (SAS Inc., Cary, NC, USA) in this study^21,22^.

### Ethical statement

Approval for the use of confidential and electronically processed patient data was granted by the Institutional Review Board of Kyung Hee University Medical Center and the Uppsala Monitoring Centre (WHO Collaborating Centre).

## Results

### Overall analysis

**Figure 1:**
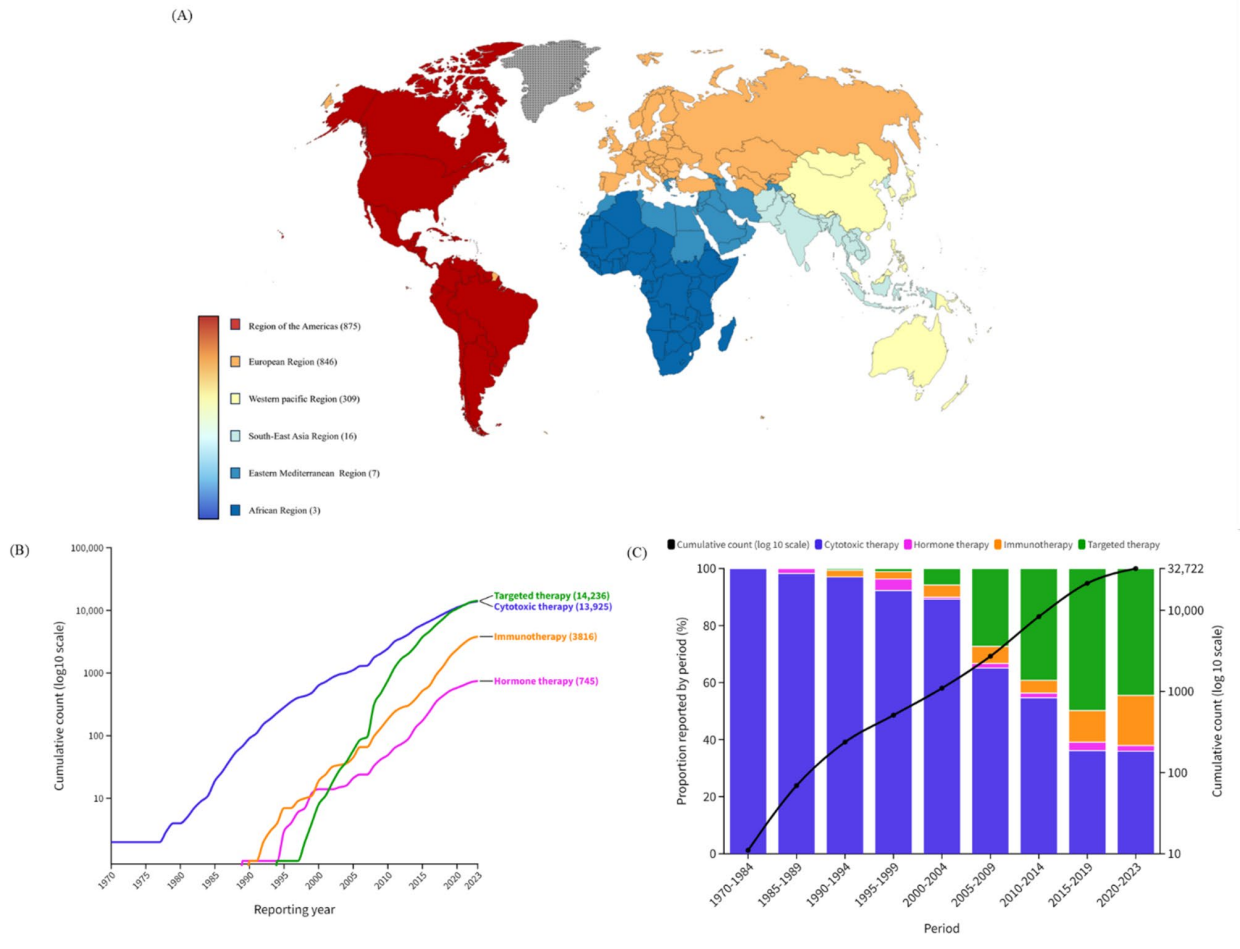
World map (**A**) and cumulative counts (**B** and **C**) of AKI cases per year in association with different anticancer drugs. AKI, acute kidney injury.

Of the 131,255,418 reports in the full database (Table 1), 32,722 reports (male, n = 17,829) and 2056 reports (male, n = 1293) of AKI and TIN, respectively, in the Vigibase database between 1967 and 2023 were identified. As depicted in Figure 1, the American region accounted for nearly half of the reports, followed by European, Western Pacific, Southeast Asia, Eastern Mediterranean, and African regions. Targeted therapy (43.5%) and cytotoxic therapy (42.6%) accounted for the highest number of reported cases of AKI, while immunotherapy (48.1%) was associated with the highest number of reported cases of TIN. The majority of reports for both AKI and TIN occurred in the age group of over 65 years (42.6% and 41.2%, respectively). The median time from initiation of the offending drug to onset was approximately 16 days for AKI and 27 days for TIN. The rate of fatal outcomes was 23.6% for AKI and 16.3% for TIN.

**Table 1:** Overall baseline characteristics of cases of anticancer drugs-associated AKI and TIN in the VigiBase, a WHO pharmacovigilance database, between 1967 and 2023.

Variables	AKI (n = 32,722)	TIN (n = 2056)
Region reporting, n (%)		
African Region	15 (0.1)	3 (0.2)
Region of the Americas	16,786 (51.3)	875 (42.6)
South-East Asia Region	361 (1.1)	16 (0.8)
European Region	11,910 (36.4)	846 (41.2)
Eastern Mediterranean Region	75 (0.2)	7 (0.3)
Western Pacific Region	3575 (10.9)	309 (15.0)
Reporting year, n (%)		
1969–1979	4 (0.01)	0 (0.00)
1980–1989	65 (0.2)	0 (0.00)
1990–1999	440 (1.3)	3 (0.2)
2000–2009	2201 (6.7)	61 (3.0)
2010–2019	18,813 (57.5)	1092 (53.1)
2020–2023	11,199 (34.2)	900 (43.8)
Reporter qualification, n (%)		
Health Professional	29,049 (88.8)	1924 (93.6)
Non-Health Professional	2231 (6.8)	64 (3.1)
Unknown	1442 (4.4)	68 (3.3)
Studies, n (%)		
Study related	12,230 (37.4)	383 (18.6)
Non-study related	20,241 (61.9)	1659 (80.7)
Unknown	251 (0.8)	14 (0.7)
Sex, n (%)		
Male	17,829 (54.5)	1293 (62.9)
Female	12,384 (37.9)	661 (32.2)
Unknown	2509 (7.7)	102 (5.0)
Age, years, n (%)		
< 18	866 (2.7)	54 (2.6)
18–44	2164 (6.6)	319 (15.5)
44–64	9603 (29.4)	530 (25.8)
≥ 65	13,952 (42.6)	847 (41.2)
Unknown	6137 (18.8)	306 (14.9)
Delay (TTO), days, mean (SD)	16.53 (80.1)	27.97 (98.1)
Drug class, n (%)		
Cytotoxic therapy	13,925 (42.6)	592 (28.8)
Hormone therapy	745 (2.3)	33 (1.6)
Immunotherapy	3816 (11.7)	989 (48.1)
Targeted therapy	14,236 (43.5)	442 (21.5)
Fatal outcomes, n (%)		
Recovered/recovering	14,561 (44.5)	1053 (51.2)
Fatal	7719 (23.6)	334 (16.3)
Unknown	10,442 (31.9)	669 (32.5)
Single drug suspected, n (%)	32,518 (99.4)	2024 (98.4)

AKI, acute kidney injury; SD, standard deviation; TIN, tubulointerstitial nephritis; TTO, time to onset; WHO, World Health Organization.

### Cumulative report analysis

**Figure 2:**
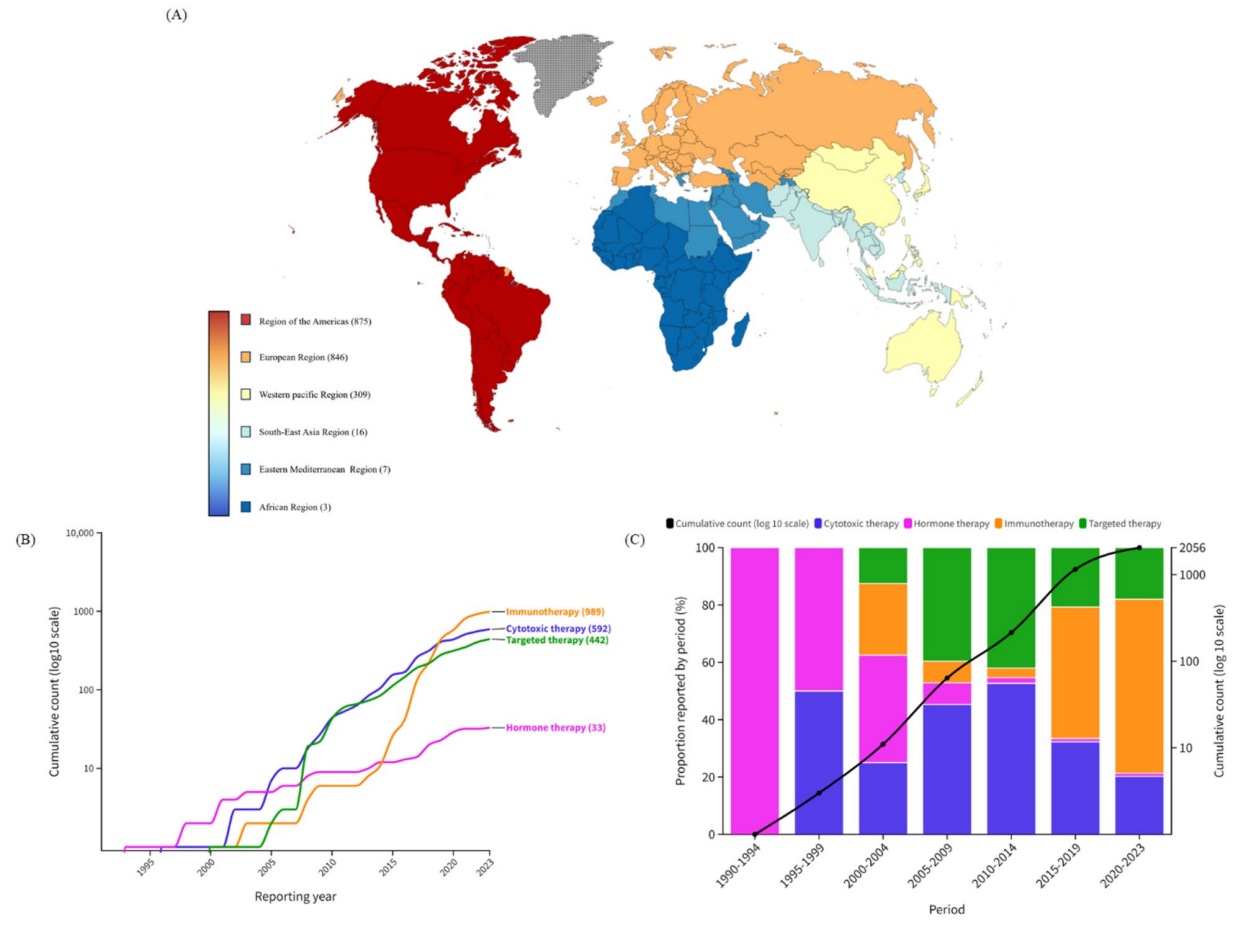
World map (**A**) and cumulative counts (**B** and **C**) of TIN cases per year in association with different anticancer drugs. TIN, tubulointerstitial nephritis.

The number of reports has seen a dramatic increase since 2010 (Figs. 1 and 2). While cytotoxic therapy has consistently been reported as the leading cause of AKI since the 1970s, the advent of targeted therapy in the early 2000s led to a steep rise in AKI attributed to targeted therapy, particularly evident in the early 2010s (Fig. 1B and C). In contrast, reports of TIN were predominantly associated with hormone therapy in the 1990s. However, following the development of immunotherapy, there has been a notable surge in TIN reports since the 2010s, with hormone therapy only accounting for a minimal number of TIN reports at present (Fig. 2B and C).

### Disproportionality analysis of AKI and TIN reports by anticancer therapies

The analysis of anticancer therapy-associated AKI and TIN reports indicated that most anticancer drugs are associated with AKI and TIN (Tables 2 and S1). Immunotherapy was associated with the most AKI reports (ROR: 8.92 [95% CI, 8.63–9.21]; IC: 3.11 [IC_0.25_: 3.06]), followed by cytotoxic therapy (ROR: 7.14 [95% CI, 7.01–7.26]; IC: 2.71 [IC_0.25_: 2.68]), targeted therapy (ROR: 5.83 [95% CI, 5.73–5.93]; IC: 2.42 [IC_0.25_: 2.40]), and hormone therapy (ROR: 2.59 [95% CI, 2.41–2.79]; IC: 1.37 [IC_0.25_: 1.24]). Similarly, immunotherapy has been reported as the most strongly associated factor with TIN (ROR: 21.74 [95% CI, 20.39–23.18]; IC: 4.34 [IC_0.25_: 4.24]), followed by cytotoxic therapy (ROR: 2.60 [95% CI, 2.40–2.82]; IC: 1.35 [IC_0.25_: 1.21]), and targeted therapy (ROR: 1.54 [95% CI, 1.40–1.69]; IC: 0.61 [IC_0.25_: 0.45]). Hormone therapy was found to have no significant association with TIN.

**Table 2:** Disproportionality analysis of anticancer-associated AKI and TIN cases.

	Total	AKI			TIN		
		Observed	ROR (95% CI)	IC (IC_0.25_)	Observed	ROR (95% CI)	IC (IC_0.25_)
Sex							
Male	2,012,633	17,829	**6.71 (6.60–6.83)**	**2.42 (2.40)**	1293	**4.56 (4.30–4.84)**	**1.98 (1.89)**
Female	2,228,455	12,384	**8.14 (7.98–8.30)**	**2.75 (2.72)**	661	**3.62 (3.34–3.93)**	**1.75 (1.62)**
Anticancer drugs							
Cytotoxic therapy	1,771,906	13,925	**7.14 (7.01–7.26)**	**2.71 (2.68)**	592	**2.60 (2.40–2.82)**	**1.35 (1.21)**
Hormone therapy	240,728	745	**2.59 (2.41–2.79)**	**1.37 (1.24)**	33	1.05 (0.74–1.47)	0.06 (-0.52)
Immunotherapy	367,813	3816	**8.92 (8.63–9.21)**	**3.11 (3.06)**	989	**21.74 (20.39–23.18)**	**4.34 (4.24)**
Targeted therapy	2,211,589	14,236	**5.83 (5.73–5.93)**	**2.42 (2.40)**	442	**1.54 (1.40–1.69)**	**0.61 (0.45)**

AKI, acute kidney injury; CI, confidence interval; IC, information component; ROR, reported odds ratio; TIN, tubulointerstitial nephritis.

Number in bold indicates statistical significance (*P* < 0.05).

Overall, a sex disproportion has been observed for both, AKI and TIN. Females showed slightly higher association with AKI (male IC [IC_0.25_]: 2.42 [2.40], female IC [IC_0.25_]: 2.75 [2.72]), while males appeared to have a slightly higher association with TIN (male IC [IC_0.25_]: 1.98 [1.89], female IC [IC_0.25_]: 1.75 [1.62]). However, the degree of association between males and females varied widely across different age groups (Tables 2 and 3). Similar associations were observed between anticancer drug and AKI and TIN (Table S2 and S3).

**Table 3:** Subgroups analysis of anticancer-associated AKI and TIN cases.

	IC (IC_0.25_) based on age, years							
	AKI				TIN			
	0–17 year	18–44 years	45–64 years	≥ 65 years	0–17 year	18–44 years	45–64 years	≥ 65 years
Sex								
Male	**4.28 (4.14)**	**2.95 (2.86)**	**2.32 (2.27)**	**1.92 (1.88)**	**2.88 (2.41)**	**3.48 (3.27)**	**1.59 (1.41)**	**1.63 (1.49)**
Female	**3.27 (3.06)**	**3.42 (3.32)**	**2.76 (2.70)**	**2.19 (2.15)**	− 0.48 (− 2.24)	**2.45 (2.08)**	**1.61 (1.38)**	**1.52 (1.32)**
Anticancer drugs								
Cytotoxic therapy	**3.92 (3.80)**	**3.33 (3.24)**	**2.47 (2.42)**	**2.15 (2.10)**	**2.41 (1.96)**	**3.12 (2.87)**	**0.83 (0.56)**	**0.63 (0.34)**
Hormone therapy	NA	**1.38 (0.65)**	**0.83 (0.47)**	**1.11 (0.96)**	NA	1.10 (− 0.97)	− 0.62 (− 2.69)	0.40 (− 0.28)
Immunotherapy	**3.01 (2.59)**	**3.28 (3.08)**	**3.44 (3.35)**	**2.85 (2.77)**	NA	**4.35 (4.00)**	**4.48 (4.28)**	**4.59 (4.44)**
Targeted therapy	**4.26 (3.96)**	**2.98 (2.83)**	**2.58 (2.53)**	**2.03 (1.99)**	NA	**2.35 (1.87)**	**0.63 (0.30)**	**0.58 (0.35)**
Total								
IC (IC_0.25_)	**7.62 (7.51)**	**5.52 (5.44)**	**4.54 (4.50)**	**4.38 (4.35)**	**5.33 (4.88)**	**5.44 (5.26)**	**3.61 (3.47)**	**3.94 (3.83)**
ROR (95% CI)	**18.34 (17.03–19.76)**	**10.54 (10.08–11.03)**	**7.49 (7.32–7.67)**	**5.31 (5.21–5.41)**	**4.42 (3.36–5.82)**	**10.60 (9.42–11.93)**	**3.49 (3.19–3.82)**	**3.64 (3.38–3.92)**

AKI, acute kidney injury; CI, confidence interval; IC, information component; ROR, reported odds ratio; TIN, tubulointerstitial nephritis.

Number in bold indicates statistical significance (*P* < 0.05).

From an age-specific perspective, reports associated with AKI were most prevalent in the age group of 0–17 years (IC [IC_0.25_]: 7.62 [7.51]), followed by the age groups of 18–44 years (IC [IC_0.25_]: 5.52 [5.44]), 45–64 years (IC [IC_0.25_]: 4.54 [4.50]), and over 65 years (IC [IC_0.25_]: 4.38 [4.35]). In contrast, reports associated with TIN were most significant in the age group of 18–44 years (IC [IC_0.25_]: 5.44 [5.26]), followed by the age groups of 0–17 years (IC [IC_0.25_]: 5.33 [4.88]), over 65 years (IC [IC_0.25_]: 3.94 [3.83]), and 45–64 years (IC [IC_0.25_]: 3.61 [3.47]). Cytotoxic therapy, immunotherapy, and targeted therapy exhibited association with AKI and TIN across all age groups, whereas hormone therapy showed no association with TIN across all age groups.

### Top anticancer drugs associated with kidney-related adverse reactions

**Figure 3:**
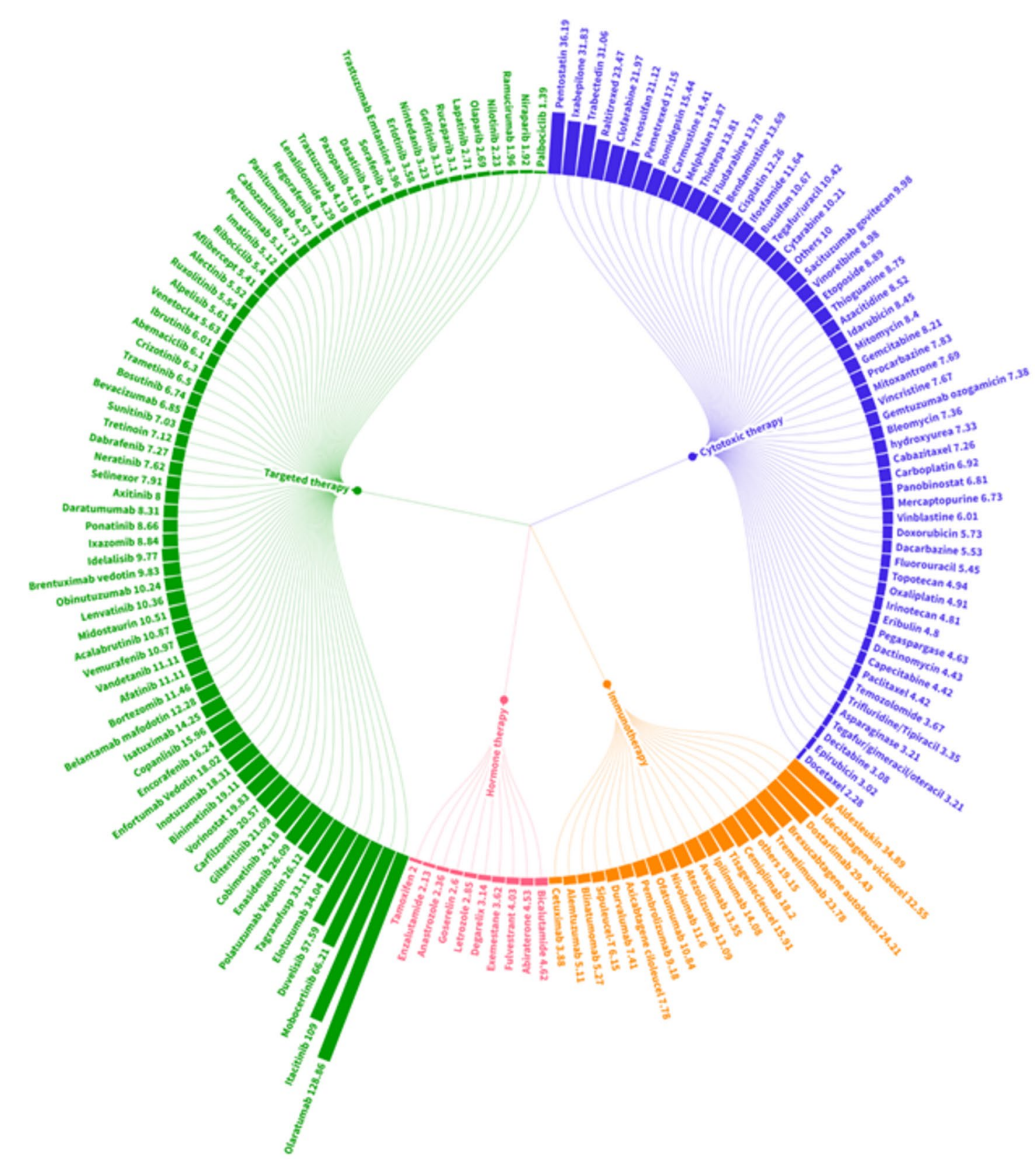
ROR of AKI cases in association with all specific medications categorized under 4 classes of anticancer drugs. AKI, acute kidney injury; ROR, reported odds ratio.

The disproportionality analysis of AKI cases associated with all specific medications is depicted in Fig. 3 and Table S1. Among conventional cytotoxic therapy agents, pentostatin (ROR: 36.19; IC_0.25_: 3.01), ixabepilone (ROR: 31.83; IC_0.25_: 3.81) and trabectedin (ROR: 31.06; IC_0.25_: 4.48) exhibited significantly elevated ROR and IC_0.25_ values. Indicating substantial disproportionality. Among all anticancer drug classes, targeted therapy showed the highest number of associated specific medications. Especially medications such as olaratumab (ROR: 128.86; IC_0.25_: 5.30), itacitinib (ROR: 109.00; IC_0.25_: 3.20), mobecertinib (ROR: 66.21; IC_0.25_: 3.41), and duvelisib (ROR: 57.59; IC_0.25_: 3.01) showed significant disproportionality. Immunotherapy emerged as another notable factor contributing to the increasing incidence of AKI cases associated with anticancer drugs. Aldesleukin, a recombinant form of human interleukin (IL)-2 (ROR 34.89; IC_0.25_: 4.45), and chimeric antigen receptor (CAR) T cell therapy drugs such as idecabtagene vicleucal (ROR: 32.55; IC_0.25_: 3.03), brexucabtagene autoleucel (ROR: 24.21; IC_0.25_: 2.74), and tisagenlecleucel (ROR 15.91; IC_0.25_: 2.74) were prominently represented among the top disproportionality signals within the immunotherapy class. Additionally, PD-1 inhibitors such as dostarlimab (ROR 29.43; IC_0.25_: 2.97), and CTLA-4 inhibitors such as tremelimumab (ROR 23.78; IC_0.25_: 2.81) showed significant associations with AKI.

**Figure 4:**
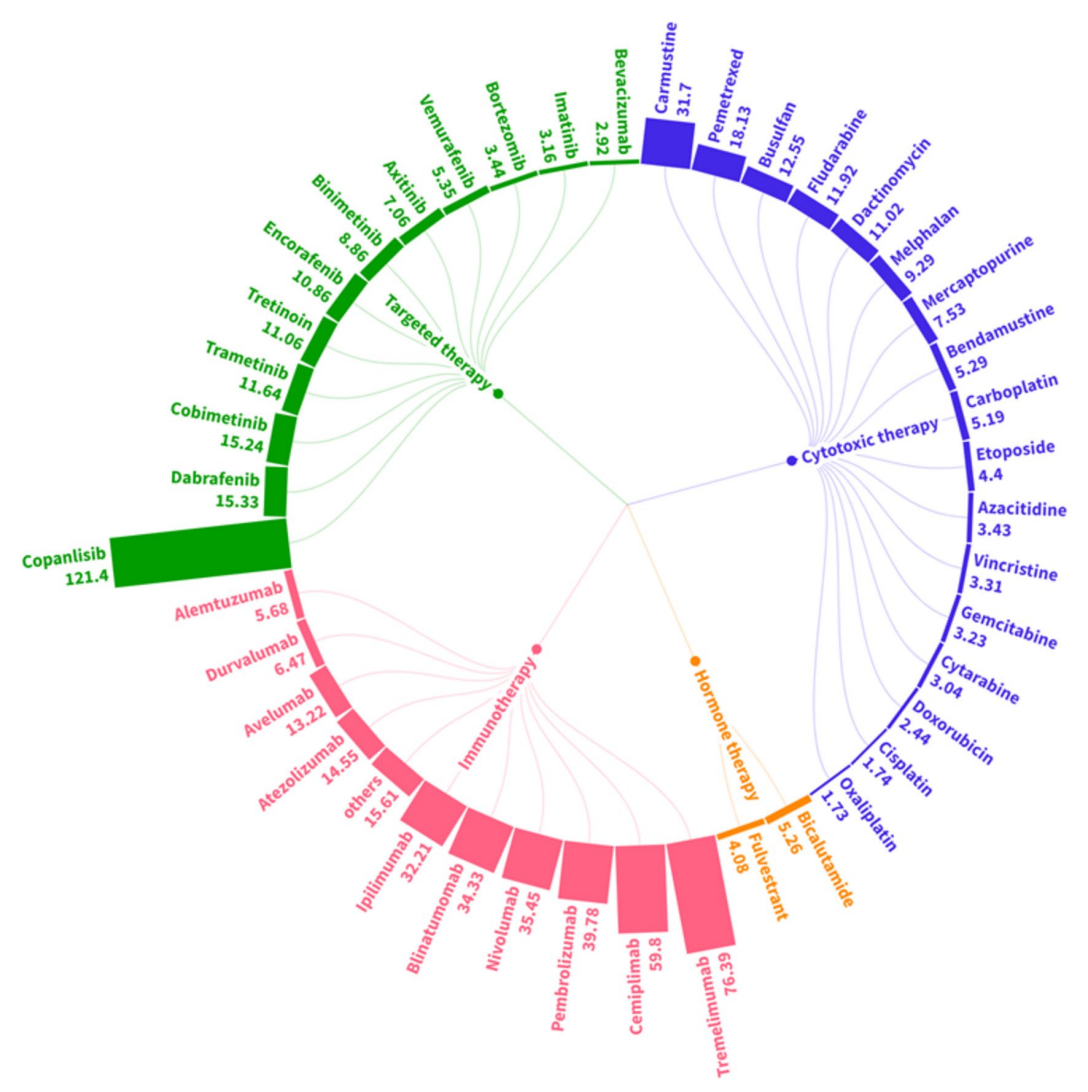
ROR of TIN cases in association with all specific medications categorized under 4 classes of anticancer drugs. ROR, reported odds ratio; TIN, tubulointerstitial nephritis.

In the case of TIN, fewer cases were reported compared to AKI, resulting in a limited number of drugs showing significant signals (Fig. 4 and Table S1). Among the four classes of anticancer drugs, immunotherapy had the largest number of medications showing a strong association, with ROR values exceeding 30. Tremelimumab (ROR: 76.39; IC_0.25_: 1.72), cemiplimab (ROR: 59.80; IC_0.25_: 3.58), pembrolizumab (ROR: 39.78; IC_0.25_: 5.02), nivolumab (ROR: 35.45; IC_0.25_: 4.87), blinatumomab (ROR: 34.33; IC_0.25_: 3.91), and ipilimumab (ROR: 32.21; IC_0.25_: 4.55) exhibited ROR values exceeding 30, indicating a substantial association with AKI. However, there were no CAR-T cell therapies that exhibited a significantly high disproportionality signal in TIN. Notably, carmustine (ROR: 31.70; IC_0.25_: 2.46) among cytotoxic therapy, as well as copanlisib (ROR: 121.40; IC_0.25_: 1.78) among targeted therapy, showed a significant association with AKI.

## Discussion

### Key findings

In this study, we conducted a large-scale, long-term investigation into the prevalence of all anticancer-associated AKI and TIN, utilizing worldwide data from the WHO international pharmacovigilance database. Since the 2010s, reports of anticancer therapy-associated AKI and TIN have markedly increased, attributed to a sharp rise in reports linked to targeted therapy and immunotherapy. It was evident that almost every drug class could potentially induce AKI and TIN, with immunotherapy being particularly associated with both AKI and TIN, followed by cytotoxic therapy, targeted therapy, and hormone therapy in the case of AKI, and followed by cytotoxic therapy and targeted therapy in the case of TIN. Interestingly, it was observed that immunotherapy displayed a more significant association with AKI and TIN compared to cytotoxic therapy, which is already well-known for its nephrotoxicity. Among the immunotherapy agents, CAR-T cell therapy showed a stronger association with AKI than with TIN. In contrast to previous findings suggesting that hormone therapy contributes to an increased risk of AKI^23^, our study revealed that hormone therapy did not exhibit a significant association with AKI compared to other drug classes, nor did it show any association with TIN. Unlike most AKI cases attributed to physiological causes occurring more commonly in the older population, anticancer therapy-associated AKI and TIN were more prevalent in younger age groups. Additionally, the fatality rates for anticancer therapy-associated AKI and TIN were found to be 23.6% and 16.3%, respectively. Considering the higher fatality rates and increased occurrence in younger age groups compared to AKI caused by other etiologies, careful consideration is warranted in selecting safer medications for patients who are expected to be more vulnerable to AKI or TIN when choosing anticancer drugs.

### Plausible underlying mechanisms

Immunotherapy effectively counteracts immune evasion of tumor cells as it enhances the pro-inflammatory process against the tumor^24^. However, this process involves interactions with the immune system, leading to certain adverse events through various mechanisms. Among them, IL-2, including aldesleukin, has the potential to trigger a severe capillary leak syndrome, leading to edema, depletion of plasma volume, and a reversible decline in glomerular filtration rate, which is primarily attributed to hypovolemia and can therefore result in AKI^25^. This provides insight into why aldesleukin shows a stronger correlation with AKI compared to TIN. Furthermore, the majority of immune checkpoint inhibitors (ICPi) operate by suppressing down-regulatory immune pathways, aiming to augment the anti-tumor immune response^26^. Previous studies have revealed that the majority of lesions in patients with ICPi- induced AKI detected through biopsy were acute TIN^24,27^. This finding aligns with our observation that ICPi exhibits particularly strong association with TIN. ICPi-induced TIN is characterized by severe inflammation involving infiltrates of inflammatory cells, with or without granuloma formation, primarily mediated by cell-mediated immunity^28^. It is assumed that ICPi reactivate drug-specific T cells and generate auto-reactive T cells and autoantibodies, which then target tubular epithelial cells, mesangial cells, and podocytes^29^. These processes contribute to the development of both AKI and TIN as consequences of ICPi treatment. Furthermore, CAR-T therapy, a type of immunotherapy, involves introducing the patient's T cells that specifically target tumor antigens. This process often results in a significant release of cytokines, leading to cytokine release syndrome. Cytokine-mediated capillary leak subsequently causes intravascular volume depletion, resulting in AKI^30^. This explains why CAR-T therapy shows a particularly significant association with AKI.

Within the realm of targeted therapy, certain medications act by inhibiting factors contributing to vascular formation, such as platelet-derived growth factor and vascular endothelial growth factor, thereby disrupting tumor angiogenesis^31^. For instance, VEGF receptor antibodies interfere with normal glomerular function and the integrity of the glomerular basement membrane, leading to AKI^32^. Additionally, phosphoinositide 3-kinase phosphorylates mammalian target of rapamycin^33^, which plays a pivotal role in signaling renal regeneration and repair in tubular cells and interstitial fibroblasts^34^. Consequently, inhibitors of phosphoinositide 3-kinase or mammalian target of rapamycin disrupt these processes, contributing to the occurrence of AKI. Interestingly, certain targeted therapies exhibited stronger association with AKI and TIN. For instance, olaratumab, a platelet-derived growth factor receptor antibody, may lead to AKI by preventing angiogenesis and kidney development, as PDGF signaling is crucial for these processes^35^. Copanlisib, a PI3-kinase inhibitor, may be associated with TIN by blocking the PI3Kγ-Akt pathway, which plays a protective, antiapoptotic role in the kidney, thus leading to increased apoptosis and accelerated renal tubular cell death^36^.

### New aspects and comparison with previous research

AKI is typically more prevalent in older individuals due to age-related declines in kidney function, rendering them more vulnerable to injury^37^. However, our findings indicate that AKI shows stronger associations in younger age groups. A previous study has linked the occurrence of AKI in critically ill children and young adults to poor outcomes, often resulting in increased mortality rates^38^. This suggests that AKI occurring in younger age groups can result in poorer prognosis.

While hormone therapy showed an association with AKI, it exhibited no association with TIN. Additionally, AKI showed significantly lower association compared to other drug classes. However, previous studies have reported an increased risk of AKI occurrence with hormone therapy like androgen deprivation therapy^23,39^. While our study primarily investigated the relationship between various anticancer treatments and AKI, hormone therapy has also been of interest due to its potential association with AKI. Previous research have proposed that hormone therapies, particularly androgen deprivation therapy, might lead to metabolic changes, subsequently reducing glomerular function. Additionally, testosterone has been thought to have a protective effect on renal function by inducing vasodilation of renal vessels^23^.

In our findings, although hormone therapy showed an association with AKI, the risk of occurrence was markedly lower compared to other drug classes, and the number of reports was minimal, indicating relatively fewer kidney-related side effects.

### Clinical and policy implications

Considering kidney-related ADRs when selecting anticancer therapy is crucial for patient mortality and safety. Cancer patients, especially those combining various medications, such as proton pump inhibitors or nonsteroidal anti-inflammatory drugs, are more vulnerable to AKI or TIN^24^. Our findings indicate that precautions should be taken when administering immunotherapy to patient groups that are expected to be more susceptible to AKI or TIN, given the higher association between immunotherapy and these ADRs compared to the nephrotoxicity caused by traditional chemotherapy. Since AKI due to ICPi is associated with increased rates of chronic kidney disease and mortality, extra vigilance is necessary in groups with a low baseline glomerular filtration rate^38^. In the event of AKI, drug discontinuation is a sensible approach. However, when TIN is suspected, there is an ongoing debate regarding whether a biopsy is necessary or if empirical treatment with corticosteroids is sufficient^40^. Some studies suggest that early corticosteroid use is associated with higher odds of kidney function recovery, highlighting the need for appropriate decision-making based on the individual patient's condition^27^. Our study revealed that the median time to onset for AKI and TIN was 16 days and 27 days, respectively, following the administration of anticancer agents. This finding underscores the critical importance of thorough monitoring and the implementation of effective therapeutic interventions in the weeks following the initiation of anticancer treatment. Healthcare professionals should remain proactive in identifying and managing these ADRs to ensure the safety of cancer patients undergoing treatment.

### Strengths and limitations

This study has several limitations. Firstly, we were unable to ascertain the exact denominator of patients exposed to anticancer drugs, which precluded the calculation of actual incidence rates. Therefore, all values are expressed in relative terms, considering that VigiBase aggregates global data, enabling generalization of the findings. Given the limitation in relative reporting and the higher reporting rates in regions such as the Americas, including the United States, and Europe, where easier access to new anticancer drugs is possible, it could be suggested that our study's global distribution pattern also reflected this trend. Although Vigibase encompasses reports from more than 170 countries worldwide, there could be bias between countries due to underreporting in some regions. AKI is known to be more prevalent among Black individuals compared to other races due to various factors, including genetic and socioeconomic risks^41^. Therefore, cautious interpretation of the data is necessary, as there could be potential bias between races in the reported cases.

Secondly, among the kidney-related ADRs which we focused on, such as TIN, they can only be verified by a diagnostic kidney biopsy. However, data related to renal biopsy results were not consistently included in the WHO's reporting system, making it changing to determine the exact cause of kidney injury. Advancements in diagnostic tools and increased awareness among medical professionals may have contributed to the rise in TIN reports since 2000, compared to AKI, which has been more easily detectable and reported since the 1970s^42,43^. Thus, as the diagnostic process for TIN is more complex than AKI, it can lead to underdiagnosis, especially in countries with limited medical resources.

Thirdly, preexisting kidney diseases were not collected in the VigiBase database, as only certain drug information and related events were mentioned. Individuals undergoing anticancer treatment often receive various drugs alongside their primary therapy, which may act as AKI inducers. NSAIDs, antimicrobial agents, and certain antibiotics combinations can all contribute to the development of AKI through various mechanisms^44,45^. Furthermore, previous studies have reported that the co-administration of proton pump inhibitors with immune checkpoint inhibitors is a high-risk factor for sustained AKI^46^. Other medications, such as diuretics, angiotensin-converting enzyme inhibitors, and angiotensin-receptor blockers, are also associated with AKI^47^. The effect of these commonly co-administered drugs should be considered as an important confounding factor that may potentially lead to bias in our findings. Although most reports in VigiBase were of single drug suspects, minimizing the influence of non-anticancer medications, we cannot exclude the possibility of bias due to other drugs.

Fourthly, this pharmacovigilance analysis did not categorize the reports based on specific cancer types for each anticancer drug. Also, the increasing use of combination therapies and the prolonged administration of anticancer drugs have made it challenging to thoroughly analyze individual drugs. However, single drugs accounted for close to 99% of AKI and TIN reports (Table 1), thus, we believe that this aspect may not have a significant impact. In addition, given that malignant tumors themselves pose a risk factor for kidney injury^48^, further research is essential to determine the relative risk of anticancer drugs for ADRs. Due to the lack of data on the number of comorbidities and concomitant prescribed drugs, which can significantly impact kidney function, underscores the need for further investigation. Moreover, our analysis reported high mortality rates due to AKI and TIN following anticancer treatment. However, due to the limitations of VigiBase as a spontaneous reporting database, we acknowledge the potential bias in our results and the need for careful interpretation, as we could not exclude all cases where death may not have been directly caused by AKI and TIN or may have been influenced by underlying malignancies.

Lastly, VigiBase is established as a spontaneous reporting system and relies on disproportional analyses vulnerable to quantitative evaluation. Although VigiBase is thoroughly managed within the WHO-UMC system, reporting bias exists and several strategies have been used to mitigate it. We utilized two metrics, IC with IC_0.25_ and ROR with 95% CI, to cross-validate the association of anticancer drugs with AKI and TIN. When anticancer drugs were used singly, adverse effects of AKI were reported at 99.4% and TIN at 98.4%, with TTO information enhancing the reliability and causality of the reported data. Additionally, we conducted an analysis using only reports from health professionals and found consistent results across all findings. Despite these efforts, unpredictable bias cannot be totally ruled out, but robust methodologies and extensive supplementary analyses are expected to minimize the impact of such biases. Further research, including prospective cohort studies, is necessary to substantiate our findings.

Despite these limitations, this study possesses significant strengths due to its utilization of large-scale, long-term data from the WHO VigiBase. Contrary to previous studies that analyzed only certain anticancer drug classes, our study examined the adverse effects of AKI and TIN for all drug classes, enabling comparisons among anticancer drugs. Additionally, by examining the associations of individual medications, we could identify which drugs within the same class are most closely related to kidney-related adverse effects. Furthermore, by scrutinizing the frequency of adverse events based on sex and age, we identified differences from the commonly known risk factors for AKI, emphasizing the need for careful monitoring even in young age groups, where AKI is generally less expected when administering anticancer drugs. This large-scale survey based on the WHO database will assist healthcare workers in selecting the most appropriate and safe medications for patients when administering anticancer drugs.

## Conclusions

Our study, utilizing data from the WHO, revealed a significant increase in kidney-related ADRs, particularly AKI and TIN, since 2010, primarily attributed to targeted therapy and immunotherapy. Immunotherapy displayed the strongest association with both AKI and TIN, followed by cytotoxic therapy, targeted therapy, and hormone therapy for AKI, and cytotoxic therapy and targeted therapy for TIN. While most drugs were linked to AKI and TIN, hormone therapy showed no association with TIN and had the lowest association with AKI compared to other drug classes. Regarding age, younger age groups, especially those under 45, exhibited a higher association with AKI and TIN, which contrasts with the common association of AKI with older age. The onset of ADRs typically took a few weeks to manifest, with fatality rates of 23.6% for AKI and 16.3% for TIN. This study underscores the significance of kidney-related ADRs, as they can also be fatal in younger age groups. These findings will aid in the selection of suitable and safe anticancer medications for individuals while also providing more precise information regarding kidney-related ADRs following cancer treatment.

### Supplementary Information

Supplementary Information.

## Data Availability

Data are available on reasonable request. Study protocol, statistical code: available from DKY (email: yonkkang@gmail.com). Dataset: available from the Uppsala Monitoring Centre or World Health Organization through a data use agreement.
