# Supplementary material for: Global burden of anticancer drug-induced acute kidney injury and tubulointerstitial nephritis from 1967 to 2023
Source: Sci Rep. 2024 Jul 12;14:16124. doi: 10.1038/s41598-024-67020-x (PMC11245615; doi:10.1038/s41598-024-67020-x)
Supplement: Supplementary file 1 — Supplementary Information. [file 41598_2024_67020_MOESM1_ESM.docx]

| **Supplementary Material** |
| --- |

**Original Paper**

**Global burden of anticancer drug-induced acute kidney injury and tubulointerstitial nephritis from 1967 to 2023**

**Running head:** Anticancer drug-induced renal adverse reactions

Soo-Young Yoon, MD, PhD^1#^, Sooji Lee, MD^2,3#^ Kyeongmin Lee, MSc^3,4#^ Jin Sug Kim, MD, PhD^1#^, Hyeon Seok Hwang, MD, PhD^1^, Andreas Kronbichler, MD, PhD^5^ Louis Jacob, MD, PhD,^6,7,8^ Ju-Young Shin, PhD,^9^ Jin A Lee, MSc,^10^ Jaeyu Park, MSc,^3,4^ Hyeri Lee, MSc,^3,4^ Hayeon Lee, MS^3,10*^, Kyunghwan Jeong, MD, PhD^1*^, Dong Keon Yon, MD, PhD, FAAAAI, FACAAI,^2,3,4,11*^

# These authors contributed equally.

* These authors contributed equally.

***Corresponding authors:**

**Dong Keon Yon,** MD, PhD, FAAAAI, FACAAI, (lead contact)

Center for Digital Health, Medical Science Research Institute Kyung Hee University Medical Center, Kyung Hee University College of Medicine, 23 Kyungheedae-ro, Dongdaemun-gu, Seoul 02447, Republic of Korea

E-mail: yonkkang@gmail.com;

**Table S1.** Disproportionality analysis of all specific medications categorized under 4 classes of anticancer drugs associated AKI and TIN.

|  | Total | | AKI | | |  | TIN | | |
| --- | --- | --- | --- | --- | --- | --- | --- | --- | --- |
|  |  |  | Observed | ROR (95% CI) | IC (IC_0.25_) |  | Observed | ROR (95% CI) | IC (IC_0.25_) |
| Cytotoxic therapy | 1,771,906 | 13,925 | | **7.14 (7.01-7.26)** | **2.71 (2.68)** |  | 592 | **2.60 (2.40-2.82)** | **1.35 (1.21)** |
| Asparaginase | 6507 | | 25 | **3.21 (2.17-4.76)** | **1.62 (0.95)** |  | 1 | 1.17 (0.16-8.32) | 0.15 (-3.63) |
| Azacitidine | 26,701 | | 270 | **8.52 (7.55-9.60)** | **3.06 (2.85)** |  | 12 | **3.43 (1.95-6.04)** | **1.64 (0.66)** |
| Bendamustine | 14,420 | | 233 | **13.69 (12.03-15.58)** | **3.71 (3.50)** |  | 10 | **5.29 (2.85-9.84)** | **2.13 (1.06)** |
| Bleomycin | 7306 | | 64 | **7.36 (5.75-9.41)** | **2.80 (2.38)** |  | 0 | NA | NA |
| Busulfan | 6086 | | 77 | **10.67 (8.52-13.36)** | **3.31 (2.93)** |  | 10 | **12.55 (6.75-23.34)** | **3.02 (1.94)** |
| Cabazitaxel | 7057 | | 61 | **7.26 (5.64-9.34)** | **2.78 (2.35)** |  | 0 | NA | NA |
| Capecitabine | 96,354 | | 508 | **4.42 (4.05-4.83)** | **2.13 (1.98)** |  | 13 | 1.03 (0.60-1.77) | 0.04 (-0.90) |
| Carboplatin | 112,010 | | 919 | **6.92 (6.48-7.39)** | **2.77 (2.66)** |  | 76 | **5.19 (4.15-6.51)** | **2.33 (1.95)** |
| Carmustine | 2174 | | 37 | **14.41 (10.41-19.95)** | **3.59 (3.04)** |  | 9 | **31.70 (16.47-61.02)** | **3.60 (2.46)** |
| Cisplatin | 131,669 | | 1890 | **12.26 (11.71-12.83)** | **3.58 (3.50)** |  | 30 | **1.74 (1.21-2.49)** | **0.78 (0.17)** |
| Cladribine | 30,857 | | 44 | 1.19 (0.88-1.60) | 0.25 (-0.26) |  | 4 | 0.99 (0.37-2.63) | -0.02 (-1.78) |
| Clofarabine | 4435 | | 114 | **21.97 (18.24-26.47)** | **4.30 (3.99)** |  | 0 | NA | NA |
| Cytarabine | 47,749 | | 577 | **10.21 (9.41-11.09)** | **3.32 (3.18)** |  | 19 | **3.04 (1.94-4.76)** | **1.53 (0.76)** |
| Dacarbazine | 5605 | | 37 | **5.53 (4.00-7.64)** | **2.38 (1.83)** |  | 2 | 2.72 (0.68-10.88) | 1.02 (-1.58) |
| Dactinomycin | 2078 | | 11 | **4.43 (2.45-8.01)** | **1.94 (0.92)** |  | 3 | **11.02 (3.55-34.20)** | **2.18 (0.11)** |
| Decitabine | 15,484 | | 57 | **3.08 (2.37-3.99)** | **1.59 (1.15)** |  | 1 | 0.49 (0.07-3.49) | -0.75 (-4.54) |
| Docetaxel | 188,131 | | 512 | **2.28 (2.09-2.48)** | **1.18 (1.03)** |  | 7 | 0.28 (0.14-0.59) | -1.75 (-3.05) |
| Doxorubicin | 112,723 | | 767 | **5.73 (5.33-6.15)** | **2.50 (2.38)** |  | 36 | **2.44 (1.76-3.38)** | **1.26 (0.70)** |
| Epirubicin | 9672 | | 35 | **3.02 (2.17-4.21)** | **1.55 (0.99)** |  | 0 | NA | NA |
| Eribulin | 3492 | | 20 | **4.80 (3.09-7.44)** | **2.13 (1.38)** |  | 0 | NA | NA |
| Etoposide | 64,304 | | 677 | **8.89 (8.24-9.59)** | **3.12 (3.00)** |  | 37 | **4.40 (3.18-6.07)** | **2.07 (1.52)** |
| Fludarabine | 19,250 | | 313 | **13.78 (12.32-15.41)** | **3.73 (3.54)** |  | 30 | **11.92 (8.33-17.05)** | **3.33 (2.72)** |
| Fluorouracil | 123,727 | | 801 | **5.45 (5.08-5.84)** | **2.43 (2.31)** |  | 14 | 0.86 (0.51-1.46) | -0.21 (-1.11) |
| Gemcitabine | 89,721 | | 872 | **8.21 (7.68-8.78)** | **3.01 (2.90)** |  | 38 | **3.23 (2.35-4.45)** | **1.65 (1.11)** |
| Gemtuzumab ozogamicin | 3298 | | 29 | **7.38 (5.12-10.64)** | **2.73 (2.11)** |  | 0 | NA | NA |
| hydroxyurea | 8936 | | 78 | **7.33 (5.87-9.16)** | **2.81 (2.43)** |  | 1 | 0.85 (0.12-6.06) | 0.58 (-2.01) |
| Idarubicin | 6866 | | 69 | **8.45 (6.67-10.72)** | **2.99 (2.59)** |  | 2 | 2.22 (0.56-8.88) | **4.89 (4.37)** |
| Ifosfamide | 19,902 | | 274 | **11.64 (10.33-13.11)** | **3.49 (3.29)** |  | 41 | **15.77 (11.60-21.43)** | 0.53 (-1.23) |
| Irinotecan | 54,530 | | 313 | **4.81 (4.31-5.38)** | **2.25 (2.06)** |  | 4 | 0.56 (0.21-1.49) | -0.77 (-11.09) |
| Ixabepilone | 869 | | 32 | **31.83 (22.36-45.30)** | **4.40 (3.81)** |  | 0 | NA | NA |
| Melphalan | 16,442 | | 269 | **13.87 (12.29-15.64)** | **3.74 (3.53)** |  | 20 | **9.29 (5.99-14.41)** | **2.95 (2.20)** |
| Mercaptopurine | 8111 | | 65 | **6.73 (5.27-8.59)** | **2.68 (2.27)** |  | 8 | **7.53 (3.76-15.06)** | **2.44 (1.23)** |
| Mitomycin | 3605 | | 36 | **8.40 (6.05-11.66)** | **2.92 (2.36)** |  | 0 | NA | NA |
| Mitoxantrone | 4481 | | 41 | **7.69 (5.65-10.46)** | **2.82 (2.30)** |  | 2 | 3.40 (0.85-13.61) | 1.20 (-1.39) |
| Nedaplatin | 16,195 | | 12 | 0.62 (0.35-1.09) | -0.67 (-1.65) |  | 0 | NA | NA |
| Oxaliplatin | 127,661 | | 746 | **4.91 (4.57-5.28)** | **2.28 (2.16)** |  | 29 | **1.73 (1.20-2.49)** | **0.77 (0.15)** |
| Paclitaxel | 144,535 | | 760 | **4.42 (4.11-4.74)** | **2.13 (2.01)** |  | 20 | 1.05 (0.68-1.64) | 0.08 (-0.68) |
| Panobinostat | 3206 | | 26 | **6.81 (4.63-10.01)** | **2.61 (1.95)** |  | 0 | NA | NA |
| Pegaspargase | 11,202 | | 62 | **4.63 (3.61-5.95)** | **2.16 (1.74)** |  | 2 | 1.36 (0.34-5.44) | 0.34 (-2.25) |
| Pemetrexed | 28,333 | | 570 | **17.15 (15.78-18.63)** | **4.05 (3.91)** |  | 67 | **18.13 (14.26-23.06)** | **4.00 (3.60)** |
| Pentostatin | 312 | | 13 | **36.19 (20.77-63.06)** | **3.95 (3.01)** |  | 1 | **24.51 (3.44-174.55)** | 1.47 (-2.31) |
| Procarbazine | 1609 | | 15 | **7.83 (4.71-13.02)** | **2.67 (1.80)** |  | 0 | NA | NA |
| Raltitrexed | 474 | | 13 | **23.47 (13.53-40.74)** | **3.66 (2.72)** |  | 0 | NA | NA |
| Romidepsin | 1428 | | 26 | **15.44 (10.47-22.76)** | **3.58 (2.93)** |  | 1 | **5.34 (0.75-37.95)** | 1.13 (-2.66) |
| Sacituzumab govitecan | 4730 | | 56 | **9.98 (7.66-12.98)** | **3.19 (2.75)** |  | 0 | NA | -1.16 (-11.49) |
| Tegafur/gimeracil/oteracil | 23,943 | | 92 | **3.21 (2.62-3.94)** | **1.66 (1.32)** |  | 2 | 0.64 (0.16-2.55) | -0.54 (-3.14) |
| Tegafur/uracil | 1051 | | 13 | **10.42 (6.03-18.02)** | **2.94 (2.00)** |  | 0 | NA | -0.35 (-10.68) |
| Temozolomide | 26,652 | | 117 | **3.67 (3.06-4.40)** | **1.85 (1.55)** |  | 6 | 1.72 (0.77-3.82) | 0.70 (-0.71) |
| Thioguanine | 1154 | | 12 | **8.75 (4.95-15.45)** | **2.73 (1.75)** |  | 0 | NA | -0.38 (-10.71) |
| Thiotepa | 3739 | | 61 | **13.81 (10.72-17.79)** | **3.62 (3.20)** |  | 1 | 2.04 (0.29-14.48) | 0.60 (-3.18) |
| Topotecan | 6440 | | 38 | **4.94 (3.59-6.80)** | **2.23 (1.69)** |  | 0 | NA | NA |
| Trabectedin | 3784 | | 136 | **31.06 (26.17-36.86)** | **4.76 (4.48)** |  | 0 | NA | NA |
| Treosulfan | 768 | | 19 | **21.12 (13.39-33.29)** | **3.78 (3.01)** |  | 0 | NA | NA |
| Trifluridine/Tipiracil | 11,981 | | 48 | **3.35 (2.52-4.45)** | **1.70 (1.23)** |  | 0 | NA | NA |
| Vinblastine | 5025 | | 36 | **6.01 (4.33-8.34)** | **2.48 (1.93)** |  | 1 | 1.52 (0.21-10.77) | 0.37 (-3.41) |
| Vincristine | 59,978 | | 546 | **7.67 (7.05-8.35)** | **2.91 (2.77)** |  | 26 | **3.31 (2.25-4.86)** | **1.66 (1.01)** |
| Vinorelbine | 10,034 | | 107 | **8.98 (7.42-10.86)** | **3.10 (2.78)** |  | 1 | 0.76 (0.11-5.39) | -0.28 (-4.06) |
| Others | 23,120 | | 274 | **10.00 (8.87-11.26)** | **3.28 (3.08)** |  | 5 | 1.65 (0.69-3.96) | 0.64 (-0.92) |
| Hormone therapy | 240,728 | | 745 | **2.59 (2.41-2.79)** | **1.37 (1.24)** |  | 33 | 1.05 (0.74-1.47) | 0.06 (-0.52) |
| Abiraterone | 36,447 | | 197 | **4.53 (3.94-5.21)** | **2.16 (1.92)** |  | 2 | 0.42 (0.10-1.67) | -1.08 (-3.67) |
| Anastrozole | 16,986 | | 48 | **2.36 (1.78-3.13)** | **1.22 (0.74)** |  | 6 | **2.69 (1.21-6.00)** | 1.25 (-0.16) |
| Bicalutamide | 5800 | | 32 | **4.62 (3.26-6.54)** | **2.12 (1.53)** |  | 4 | **5.26 (1.97-14.02)** | **1.84 (0.07)** |
| Degarelix | 3462 | | 13 | **3.14 (1.82-5.41)** | **1.54 (0.60)** |  | 0 | NA | NA |
| Enzalutamide | 60,826 | | 155 | **2.13 (1.82-2.49)** | **1.08 (0.82)** |  | 3 | 0.38 (0.12-1.17) | -1.28 (-3.35) |
| Exemestane | 11,774 | | 51 | **3.62 (2.75-4.77)** | **1.82 (1.35)** |  | 4 | 2.59 (0.97-6.90) | 1.14 (-0.63) |
| Fulvestrant | 13,084 | | 63 | **4.03 (3.14-5.16)** | **1.97 (1.55)** |  | 7 | **4.08 (1.94-8.56)** | **1.76 (0.46)** |
| Goserelin | 7065 | | 22 | **2.60 (1.71-3.95)** | **1.33 (0.61)** |  | 3 | **3.24 (1.04-10.04)** | 1.29 (-0.77) |
| Letrozole | 26,688 | | 91 | **2.85 (2.32-3.50)** | **1.49 (1.14)** |  | 3 | 0.86 (0.28-2.66) | -0.19 (-2.26) |
| Leuprorelin | 49,837 | | 52 | 0.87 (0.66-1.14) | -0.20 (-0.66) |  | 0 | NA | NA |
| Tamoxifen | 8759 | | 21 | **2.00 (1.30-3.07)** | **0.97 (0.23)** |  | 1 | 0.87 (0.12-6.18) | -0.14 (-3.92) |
| Immunotherapy | 367,813 | | 3816 | **8.92 (8.63-9.21)** | **3.11 (3.06)** |  | 989 | **21.74 (20.39-23.18)** | **4.34 (4.24)** |
| Aldesleukin | 2089 | | 84 | **34.89 (28.05-43.40)** | **4.81 (4.45)** |  | 2 | **7.30 (1.83-29.23)** | 1.69 (-0.90) |
| Alemtuzumab | 51,097 | | 311 | **5.11 (4.57-5.71)** | **2.33 (2.15)** |  | 38 | **5.68 (4.13-7.81)** | **2.42 (1.88)** |
| Atezolizumab | 20,514 | | 317 | **13.09 (11.71-14.63)** | **3.66 (3.47)** |  | 39 | **14.55 (10.62-19.92)** | **3.63 (3.10)** |
|  |  |  |  |  |  |  |  |  |  |
| Avelumab | 2310 | | 37 | **13.55 (9.79-18.75)** | **3.52 (2.97)** |  | 4 | **13.22 (4.96-35.27)** | **2.49 (0.72)** |
| Axicabtagene ciloleucel | 3348 | | 31 | **7.78 (5.46-11.08)** | **2.8 (2.20)** |  | 0 | NA | NA |
| Bcg Vaccine | 14,492 | | 17 | 0.98 (0.61-1.57) | -0.03 (-0.85) |  | 5 | **2.63 (1.09-6.32)** | 1.20 (-0.37) |
| Blinatumomab | 7149 | | 45 | **5.27 (3.93-7.07)** | **2.33 (1.83)** |  | 32 | **34.33 (24.25-48.60)** | **4.50 (3.91)** |
| Brexucabtagene autoleucel | 460 | | 13 | **24.21 (13.95-42.02)** | **3.68 (2.74)** |  | 0 | NA | NA |
| Cemiplimab | 2057 | | 44 | **18.20 (13.50-24.53)** | **3.91 (3.40)** |  | 16 | **59.80 (36.56-97.83)** | **4.42 (3.58)** |
| Cetuximab | 47,290 | | 219 | **3.88 (3.39-4.43)** | **1.94 (1.72)** |  | 3 | 0.48 (0.16-1.50) | -0.94 (-3.01) |
| Dostarlimab | 410 | | 14 | **29.43 (17.27-50.15)** | **3.87 (2.97)** |  | 0 | NA | NA |
| Durvalumab | 11,791 | | 104 | **7.41 (6.11-8.99)** | **2.83 (2.51)** |  | 10 | **6.47 (3.48-12.04)** | **2.36 (1.28)** |
| Idecabtagene vicleucel | 372 | | 14 | **32.55 (19.08-55.53)** | **3.94 (3.03)** |  | 0 | NA | NA |
| Ipilimumab | 32,569 | | 540 | **14.08 (12.93-15.33)** | **3.77 (3.63)** |  | 136 | **32.21 (27.20-38.14)** | **4.84 (4.55)** |
| Nivolumab | 79,148 | | 1081 | **11.60 (10.92-12.32)** | **3.50 (3.40)** |  | 359 | **35.45 (31.92-39.36)** | **5.05 (4.87)** |
| Ofatumumab | 2802 | | 36 | **10.84 (7.80-15.05)** | **3.24 (2.69)** |  | 0 | NA | NA |
| Pembrolizumab | 65,562 | | 712 | **9.18 (8.52-9.88)** | **3.17 (3.05)** |  | 334 | **39.78 (35.69-44.34)** | **5.20 (5.02)** |
| Sipuleucel-T | 8042 | | 59 | **6.15 (4.76-7.95)** | **2.55 (2.12)** |  | 0 | NA | NA |
| Tisagenlecleucel | 3093 | | 58 | **15.91 (12.27-20.63)** | **3.80 (3.36)** |  | 0 | NA | NA |
| Tremelimumab | 504 | | 14 | **23.78 (13.98-40.46)** | **3.71 (2.81)** |  | 5 | **76.39 (31.65-184.37)** | **3.28 (1.72)** |
| others | 2936 | | 66 | **19.15 (15.00-24.44)** | **4.05 (3.64)** |  | 6 | **15.61 (7.01-34.79)** | **2.88 (1.46)** |
| Targeted therapy | 2,211,589 | | 14,236 | **5.83 (5.73-5.93)** | **2.42 (2.40)** |  | 442 | **1.54 (1.40-1.69)** | **0.61 (0.45)** |
| Abemaciclib | 15,135 | | 110 | **6.10 (5.05-7.36)** | **2.57 (2.25)** |  | 1 | 0.50 (0.07-3.58) | -0.73 (-4.51) |
| Acalabrutinib | 1551 | | 20 | **10.87 (7.00-16.90)** | **3.12 (2.37)** |  | 0 | NA | NA |
| Afatinib | 19,844 | | 261 | **11.11 (9.83-12.55)** | **3.43 (3.22)** |  | 2 | 0.77 (0.19-3.07) | -0.31 (-2.90) |
| Aflibercept | 9598 | | 62 | **5.41 (4.22-6.95)** | **2.38 (1.96)** |  | 0 | NA | NA |
| Alectinib | 6682 | | 44 | **5.52 (4.10-7.42)** | **2.39 (1.88)** |  | 1 | 1.14 (0.16-8.10) | 0.12 (-3.66) |
| Alpelisib | 8662 | | 58 | **5.61 (4.33-7.27)** | **2.42 (1.99)** |  | 0 | NA | NA |
| Avapritinib | 5474 | | 11 | 1.68 (0.93-3.03) | 0.70 (-0.32) |  | 0 | NA | NA |
| Axitinib | 19,456 | | 185 | **8.00 (6.92-9.25)** | **2.96 (2.72)** |  | 18 | **7.06 (4.45-11.22)** | **2.60 (1.81)** |
| Belantamab mafodotin | 1858 | | 27 | **12.28 (8.39-17.95)** | **3.33 (2.69)** |  | 0 | NA | NA |
| Bevacizumab | 104,610 | | 850 | **6.85 (6.40-7.33)** | **2.75 (2.64)** |  | 40 | **2.92 (2.14-3.98)** | **1.51 (0.98)** |
| Binimetinib | 5171 | | 116 | **19.11 (15.90-22.98)** | **4.12 (3.81)** |  | 6 | **8.86 (3.98-19.73)** | **2.46 (1.05)** |
| Bortezomib | 57,750 | | 781 | **11.46 (10.68-12.30)** | **3.48 (3.37)** |  | 26 | **3.44 (2.34-5.05)** | **1.71 (1.06)** |
| Bosutinib | 9221 | | 74 | **6.74 (5.36-8.47)** | **2.69 (2.30)** |  | 0 | NA | NA |
| Brentuximab vedotin | 8231 | | 96 | **9.83 (8.04-12.02)** | **3.22 (2.88)** |  | 2 | 1.85 (0.46-7.41) | 0.66 (-1.93) |
| Cabozantinib | 37,174 | | 210 | **4.73 (4.13-5.42)** | **2.22 (1.99)** |  | 0 | NA | NA |
| Carfilzomib | 22,708 | | 546 | **20.57 (18.90-22.40)** | **4.30 (4.16)** |  | 5 | 1.68 (0.70-4.03) | 0.66 (-0.90) |
| Cobimetinib | 3509 | | 99 | **24.18 (19.80-29.53)** | **4.40 (4.07)** |  | 7 | **15.24 (7.26-32.00)** | **2.97 (1.66)** |
| Copanlisib | 319 | | 6 | **15.96 (7.11-35.79)** | **2.88 (1.47)** |  | 5 | **121.40 (50.17-293.74)** | **3.34 (1.78)** |
| Crizotinib | 16,797 | | 126 | **6.30 (5.28-7.50)** | **2.61 (2.32)** |  | 6 | **2.72 (1.22-6.07)** | 1.27 (-0.15) |
| Dabrafenib | 21,971 | | 190 | **7.27 (6.30-8.38)** | **2.83 (2.59)** |  | 44 | **15.33 (11.40-20.61)** | **3.72 (3.22)** |
| Daratumumab | 18,944 | | 187 | **8.31 (7.19-9.59)** | **3.01 (2.77)** |  | 3 | 1.21 (0.39-3.74) | 0.23 (-1.84) |
| Dasatinib | 36,520 | | 179 | **4.10 (3.54-4.75)** | **2.02 (1.77)** |  | 5 | 1.04 (0.43-2.51) | 0.06 (-1.51) |
| Duvelisib | 170 | | 11 | **57.59 (31.26-106.10)** | **4.03 (3.01)** |  | 0 | NA | NA |
| Elotuzumab | 1222 | | 48 | **34.04 (25.51-45.43)** | **4.62 (4.14)** |  | 0 | NA | NA |
| Enasidenib | 362 | | 11 | **26.09 (14.31-47.54)** | **3.62 (2.60)** |  | 0 | NA | NA |
| Encorafenib | 6326 | | 121 | **16.24 (13.57-19.45)** | **3.91 (3.61)** |  | 9 | **10.86 (5.65-20.89)** | **2.84 (1.70)** |
| Enfortumab Vedotin | 1463 | | 31 | **18.02 (12.63-25.72)** | **3.80 (3.20)** |  | 2 | **10.43 (2.61-41.77)** | 1.85 (-0.74) |
| Erlotinib | 51,620 | | 221 | **3.58 (3.14-4.09)** | **1.83 (1.60)** |  | 3 | 0.44 (0.14-1.37) | -1.05 (-3.12) |
| Gefitinib | 10,408 | | 39 | **3.13 (2.29-4.29)** | **1.60 (1.07)** |  | 2 | 1.46 (0.37-5.86) | 0.42 (-2.17) |
| Gilteritinib | 1214 | | 30 | **21.09 (14.68-30.31)** | **3.96 (3.35)** |  | 0 | NA | NA |
| Ibrutinib | 92,293 | | 659 | **6.01 (5.56-6.49)** | **2.57 (2.44)** |  | 18 | 1.49 (0.94-2.36) | 0.55 (-0.24) |
| Idelalisib | 9140 | | 106 | **9.77 (8.07-11.84)** | **3.22 (2.89)** |  | 0 | NA | NA |
| Imatinib | 77,357 | | 472 | **5.12 (4.68-5.61)** | **2.34 (2.19)** |  | 32 | **3.16 (2.23-4.47)** | **1.61 (1.02)** |
| Inotuzumab | 1719 | | 37 | **18.31 (13.22-25.37)** | **3.87 (3.32)** |  | 0 | NA | NA |
| Isatuximab | 1664 | | 28 | **14.25 (9.81-20.70)** | **3.51 (2.88)** |  | 2 | **9.17 (2.29-36.71)** | 1.80 (-0.79) |
| Itacitinib | 95 | | 11 | **109 (58.14-204.36)** | **4.23 (3.20)** |  | 0 | NA | NA |
| Ixazomib | 15,517 | | 163 | **8.84 (7.58-10.32)** | **3.10 (2.84)** |  | 1 | 0.49 (0.07-3.49) | -0.76 (-4.54) |
| Lapatinib | 16,940 | | 55 | **2.71 (2.08-3.53)** | **1.41 (0.97)** |  | 0 | NA | NA |
| Lenalidomide | 652,665 | | 3296 | **4.29 (4.15-4.44)** | **2.07 (2.02)** |  | 70 | 0.82 (0.65-1.03) | -0.29 (-0.69) |
| Lenvatinib | 29,101 | | 357 | **10.36 (9.33-11.50)** | **3.34 (3.16)** |  | 7 | 1.83 (0.87-3.85) | 0.80 (-0.51) |
| Midostaurin | 3289 | | 41 | **10.51 (7.72-14.30)** | **3.22 (2.70)** |  | 0 | NA | NA |
| Mobocertinib | 190 | | 14 | **66.21 (38.42-114.11)** | **4.32 (3.41)** |  | 0 | NA | NA |
| Neratinib | 3197 | | 29 | **7.62 (5.29-10.98)** | **2.77 (2.15)** |  | 0 | NA | NA |
| Nilotinib | 38,206 | | 102 | **2.23 (1.84-2.71)** | **1.15 (0.82)** |  | 4 | 0.80 (0.30-2.13) | -0.29 (-2.06) |
| Nintedanib | 30,036 | | 116 | **3.23 (2.69-3.87)** | **1.67 (1.37)** |  | 0 | NA | NA |
| Niraparib | 41,768 | | 96 | **1.92 (1.57-2.34)** | **0.93 (0.59)** |  | 0 | NA | NA |
| Obinutuzumab | 6341 | | 77 | **10.24 (8.18-12.82)** | **3.26 (2.88)** |  | 2 | 2.4 (0.60-9.62) | 0.91 (-1.68) |
| Ofatumumab | 0 | | 0 | NA | NA |  | 0 | NA | NA |
| Olaparib | 15,812 | | 51 | **2.69 (2.05-3.55)** | **1.40 (0.94)** |  | 1 | 0.48 (0.07-3.42) | -0.78 (-4.56) |
| Olaratumab | 388 | | 52 | **128.86 (96.21-172.57)** | **5.76 (5.30)** |  | 0 | NA | NA |
| Osimertinib | 15,623 | | 26 | 1.39 (0.94-2.04) | 0.46 (-0.19) |  | 0 | NA | NA |
| Palbociclib | 108,282 | | 181 | **1.39 (1.20-1.61)** | **0.48 (0.23)** |  | 3 | 0.21 (0.07-0.65) | -2.07 (-4.14) |
| Panitumumab | 18,701 | | 102 | **4.57 (3.76-5.55)** | **2.16 (1.83)** |  | 1 | 0.41 (0.06-2.89) | -0.98 (-4.76) |
| Pazopanib | 40,274 | | 200 | **4.16 (3.62-4.78)** | **2.04 (1.80)** |  | 8 | 1.51 (0.76-3.03) | 0.56 (-0.66) |
| Pertuzumab | 23,291 | | 142 | **5.11 (4.33-6.03)** | **2.32 (2.05)** |  | 0 | NA | NA |
| Polatuzumab Vedotin | 1348 | | 41 | **26.12 (19.14-35.64)** | **4.29 (3.77)** |  | 0 | NA | NA |
| Ponatinib | 12,829 | | 132 | **8.66 (7.29-10.28)** | **3.06 (2.77)** |  | 4 | 2.38 (0.89-6.34) | 1.04 (-0.72) |
| Ramucirumab | 13,654 | | 32 | **1.96 (1.38-2.77)** | **0.94 (0.36)** |  | 0 | NA | NA |
| Regorafenib | 23,153 | | 119 | **4.30 (3.59-5.15)** | **2.08 (1.78)** |  | 1 | 0.33 (0.05-2.34) | -1.24 (-5.02) |
| Ribociclib | 28,576 | | 184 | **5.40 (4.67-6.24)** | **2.41 (2.16)** |  | 3 | 0.80 (0.26-2.48) | -0.28 (-2.35) |
| Rucaparib | 15,612 | | 58 | **3.10 (2.4-4.02)** | **1.60 (1.17)** |  | 0 | NA | NA |
| Ruxolitinib | 89,623 | | 591 | **5.54 (5.11-6.01)** | **2.45 (2.32)** |  | 5 | 0.43 (0.18-1.02) | -1.16 (-2.72) |
| Selinexor | 4781 | | 45 | **7.91 (5.9-10.61)** | **2.87 (2.37)** |  | 0 | NA | NA |
| Sorafenib | 44,823 | | 214 | **4.00 (3.49-4.57)** | **1.98 (1.76)** |  | 5 | 0.85 (0.35-2.04) | -0.21 (-1.78) |
| Sunitinib | 53,864 | | 450 | **7.03 (6.41-7.71)** | **2.79 (2.63)** |  | 22 | 3.12 (2.05-4.74) | 1.57 (0.86) |
| Tagraxofusp | 549 | | 21 | **33.11 (21.41-51.21)** | **4.21 (3.48)** |  | 0 | NA | NA |
| Trametinib | 23,004 | | 178 | **6.50 (5.61-7.53)** | **2.67 (2.42)** |  | 35 | **11.64 (8.35-16.22)** | **3.34 (2.77)** |
| Trastuzumab | 64,178 | | 321 | **4.19 (3.75-4.68)** | **2.05 (1.87)** |  | 10 | 1.19 (0.64-2.21) | 0.24 (-0.84) |
| Trastuzumab Emtansine | 8875 | | 42 | **3.96 (2.92-5.36)** | **1.93 (1.42)** |  | 0 | NA | NA |
| Tretinoin | 4834 | | 41 | **7.12 (5.24-9.68)** | **2.72 (2.20)** |  | 7 | **11.06 (5.27-23.21)** | **2.73 (1.42)** |
| Vandetanib | 759 | | 10 | **11.11 (5.95-20.74)** | **2.90 (1.82)** |  | 2 | **20.14 (5.03-80.68)** | 2.06 (-0.53) |
| Vemurafenib | 12,846 | | 167 | **10.97 (9.42-12.78)** | **3.40 (3.14)** |  | 9 | **5.35 (2.78-10.28)** | **2.12 (0.98)** |
| Venetoclax | 52,202 | | 350 | **5.63 (5.07-6.25)** | **2.47 (2.30)** |  | 3 | 0.44 (0.14-1.36) | -1.07 (-3.14) |
| Vismodegib | 7469 | | 13 | 1.45 (0.84-2.50) | 0.51 (-0.43) |  | 0 | NA | NA |
| Vorinostat | 2751 | | 64 | **19.83 (15.48-25.41)** | **4.08 (3.67)** |  | 0 | NA | NA |

AKI, acute kidney injury; CI, confidence interval; IC, information component; ROR, reported odds ratio; TIN, tubulointerstitial nephritis.

Number in bold indicates statistical significance (P <0.05).

**Table S2.** Disproportionality analysis of anticancer-associated AKI and TIN cases reported from health professional.

|  | Total | AKI (n=29,049) | | |  | TIN (n=1924) | | |
| --- | --- | --- | --- | --- | --- | --- | --- | --- |
|  |  | Observed | ROR (95% CI) | IC (IC_0.25_) |  | Observed | ROR (95% CI) | IC (IC_0.25_) |
| **Sex** |  |  |  |  |  |  |  |  |
| Male | 1,430,352 | 15,871 | **5.01 (4.92-5.11)** | **1.93 (1.90)** |  | 1216 | **3.42 (3.21-3.65)** | **1.53 (1.43)** |
| Female | 1,550,951 | 10,911 | **5.55 (5.43-5.67)** | **2.12 (2.09)** |  | 614 | **2.56 (2.36-2.79)** | **1.23 (1.10)** |
| **Anticancer drugs** |  |  |  |  |  |  |  |  |
| Cytotoxic therapy | 1,211,499 | 12,352 | **6.41 (6.29-6.53)** | **2.51 (2.49)** |  | 547 | **2.57 (2.36-2.8)** | **1.31 (1.17)** |
| Hormone therapy | 121,710 | 605 | **2.81 (2.59-3.04)** | **1.48 (1.34)** |  | 25 | 1.13 (0.77-1.68) | 0.18 (-0.49) |
| Immunotherapy | 278,081 | 3521 | **7.39 (7.15-7.65)** | **2.83 (2.77)** |  | 945 | **20.56 (19.23-21.98)** | **4.22 (4.11)** |
| Targeted therapy | 1,601,347 | 12,571 | **4.90 (4.81-4.99)** | **2.14 (2.11)** |  | 407 | **1.42 (1.28-1.57)** | **0.49 (0.32)** |

AKI, acute kidney injury; CI, confidence interval; IC, information component; ROR, reported odds ratio; TIN, tubulointerstitial nephritis.

Number in bold indicates statistical significance (P <0.05).

**Table S3.** Subgroups analysis of anticancer-associated AKI and TIN cases reported from health professional.

|  | IC (IC_0.25_) based on age, years | | | | | | | | |
| --- | --- | --- | --- | --- | --- | --- | --- | --- | --- |
|  | AKI (n=29,049) | | | |  | TIN (n=1924) | | | |
|  | 0–17 year | 18-44 years | 45–64 years | ≥65 years |  | 0–17 year | 18-44 years | 45–64 years | ≥65 years |
| **Sex** |  |  |  |  |  |  |  |  |  |
| Male | **3.55 (3.40)** | **2.74 (2.64)** | **2.30 (2.25)** | **1.78 (1.74)** |  | **2.28 (1.81)** | **3.33 (3.12)** | **1.61 (1.42)** | **1.50 (1.35)** |
| Female | **2.45 (2.22)** | **3.08 (2.97)** | **2.57 (2.52)** | **1.94 (1.89)** |  | -0.96 (-2.73) | **2.22 (1.83)** | **1.58 (1.34)** | **1.27 (1.06)** |
| **Anticancer drugs** |  |  |  |  |  |  |  |  |  |
| Cytotoxic therapy | **3.15 (3.01)** | **3.06 (2.96)** | **2.49 (2.43)** | **1.95 (1.90)** |  | **1.85 (1.39)** | **2.97 (2.72)** | **0.92 (0.64)** | **0.32 (0.01)** |
| Hormone therapy | NA | **1.69 (0.87)** | **0.94 (0.54)** | **1.16 (0.99)** |  | NA | 1.50 (-0.57) | -0.74 (-3.33) | 0.33 (-0.46) |
| Immunotherapy | **2.55 (2.12)** | **2.87 (2.66)** | **3.23 (3.14)** | **2.52 (2.43)** |  | NA | **3.96 (3.60)** | **4.35 (4.14)** | **4.27 (4.12)** |
| Targeted therapy | **3.51 (3.20)** | **2.58 (2.42)** | **2.40 (2.34)** | **1.77 (1.73)** |  | NA | **2.09 (1.60)** | **0.50 (0.15)** | **0.33 (0.09)** |
| **Total** |  |  |  |  |  |  |  |  |  |
| IC (IC_0.25_) | **6.96 (6.84)** | **5.17 (5.09)** | **4.34 (4.31)** | **3.92 (3.89)** |  | **4.97 (4.51)** | **5.21 (5.02)** | **3.51 (3.36)** | **3.49 (3.37)** |
| ROR (95% CI) | **11.02 (10.18-11.94)** | **8.81 (8.39-9.26)** | **7.86 (7.67-8.07)** | **4.76 (4.66-4.86)** |  | **9.64 (8.52-10.90)** | **3.71 (3.37-4.09)** | **3.23 (2.99-3.50)** | **3.64 (3.38-3.92)** |

AKI, acute kidney injury; CI, confidence interval; IC, information component; ROR, reported odds ratio; TIN, tubulointerstitial nephritis.

Number in bold indicates statistical significance (P <0.05).

**Figure S1.** ROR of AKI cases in association with all specific medications categorized under 4 classes of anticancer drugs: (A) cytotoxic therapy, (B) hormone therapy, (C) immunotherapy, (D) targeted therapy. AKI, acute kidney injury; ROR, reported odds ratio.


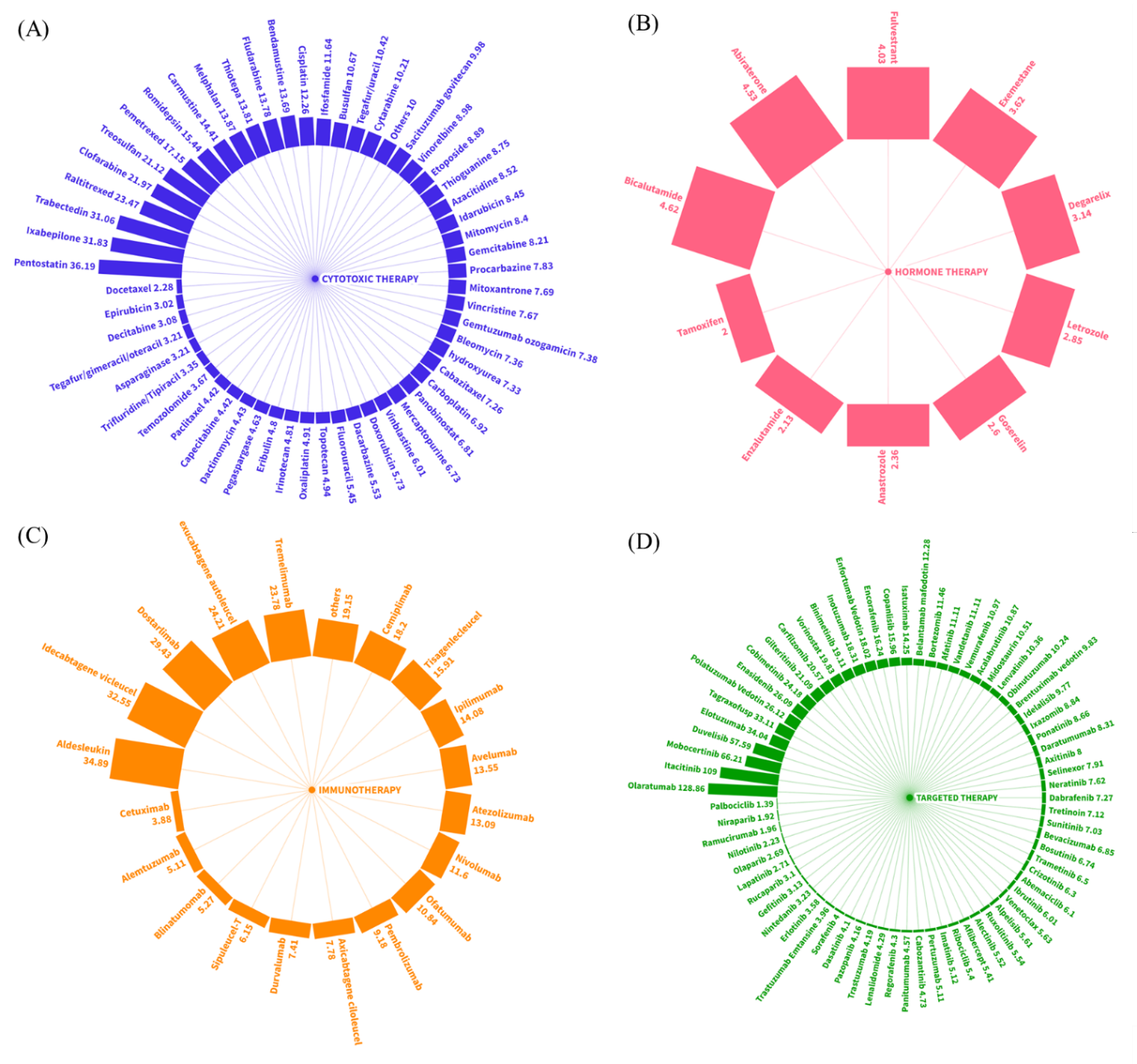


**Supplementary Methods**

***Overview***

Pharmacovigilance and medical product safety are crucial for global health. The World Health Organization (WHO) initiated the Pilot Research Project for International Drug Monitoring in 1963, which later evolved into VigiBase, coordinated by the Uppsala Monitoring Center (UMC) in Sweden. As of 2024, VigiBase has expanded to include 157 countries, 22 associates, and numerous regional reporting centers contributing individual case safety reports (ICSR). The primary objective of VigiBase is to detect, understand, evaluate, and prevent adverse effects associated with various medical products.


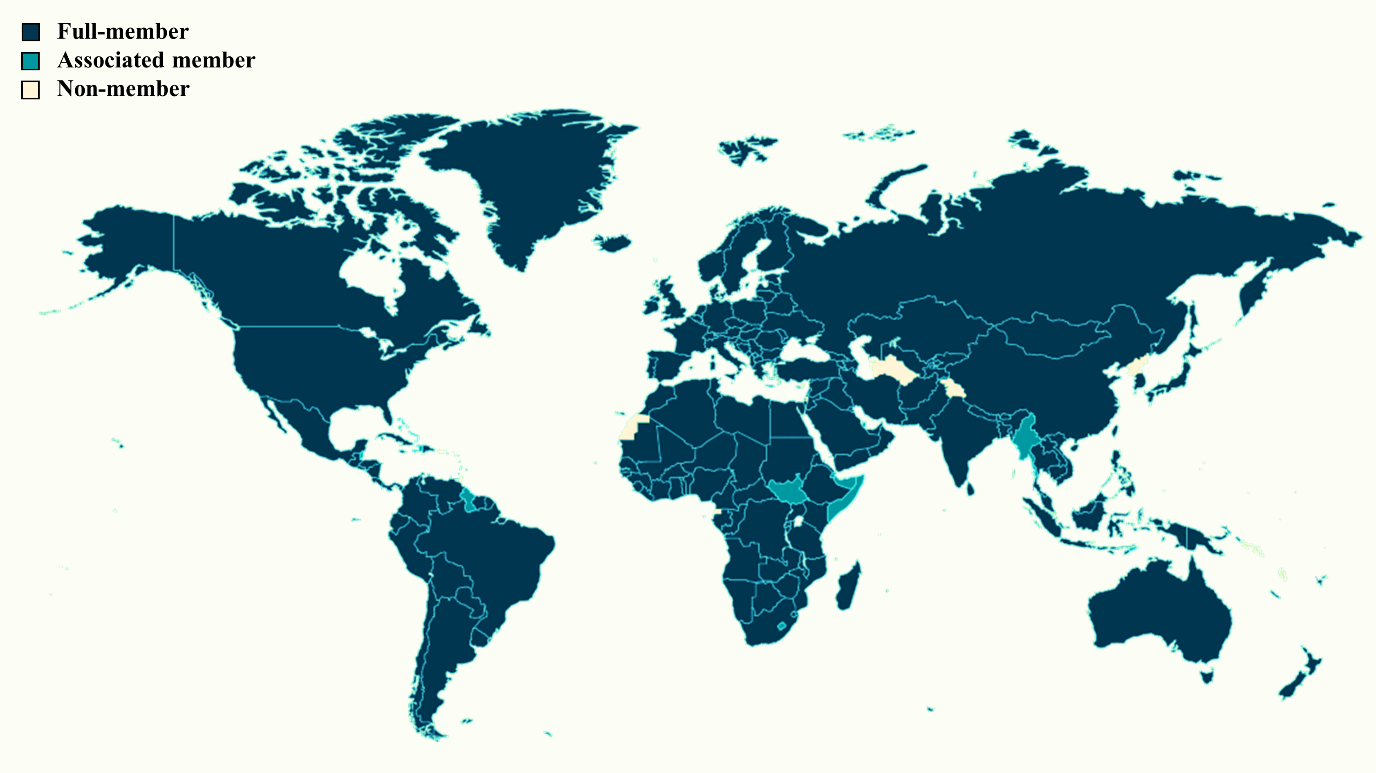


***Data source***

ICSR, sourced from VigiBase encompass a wide array of data points. These include the country of origin (categorized into six regions: Africa, Americas, Europe, Western Pacific, South-East Asia, and Eastern Mediterranean), patient demographics (age, sex, and region), reporter qualification (health professional or non-health professional), details of the reported drugs (indication, dosage, route of administration, and start dates), adverse drug reaction information classified according to the Medical Dictionary for Regulatory Activities (MedDRA) terminology (time to onset, seriousness, and fatal outcomes), and supplementary case information (concomitant medication use). The MedDRA is a hierarchical terminology system that categorizes medical terms into five distinct levels: Lowest Level Terms, Preferred Terms, High Level Terms, High Level Group Terms, and System Organ Classes. For patient privacy, all data within VigiBase are anonymized, and the policies of the WHO and UMC prohibit the publication of individual patient data.

***Signal detection***

UMC conducts regular surveillance of VigiBase to identify previously unrecognized ADRs. A qualitative screening of the scientific literature via VigiLyze, a specialized signal detection and management system, enables more focused searches and aids in identifying whether potential safety signals warrant further investigation. UMC also engages in regular monitoring of activities by international regulatory agencies and collaborates closely with external clinical expert groups to identify safety issues. The signal team conducts a case-by-case analysis of chosen drug-adverse reaction pairings. The initial assessment involves excluding other more likely causes, verifying if the adverse reaction is properly documented in the product information, and determining if the combination justifies further investigation.

***Disproportional analysis***

The information component (IC), initially introduced through the Bayesian Confidence Propagation Neural Network, is a metrics for assessing the disproportionality between the observed and the expected reporting of a drug‐ ADR pair. A positive IC value indicates that a particular drug‐ ADR pair is reported more often than expected, based on all the reports in the database. Conversely, a negative IC value means that the drug‐ADR pair is reported less frequently than expected. The higher the value of the IC, the more the combination stands out from the background. IC value used the Bayesian neural network method developed and validated by UMC. The statistical formula for calculating IC is as follows: IC = log2([Nobserved + 0.5] / [Nexpected + 0.5]), where Nexpected is the number of reports expected for the combination of vaccine and adverse effects and is calculated by [Ndrug × Neffect] / Ntotal; Nobserved indicates the number of reports for a certain adverse reaction associated with a specific vaccine. Additionally, Ndrug represents the number of reports for a certain vaccine regardless of the ADR, Neffect represents the number of reports for a given reaction regardless of the drug, and Ntotal represents the total number of reports in the database. IC0.25 refers to the lower limit of the 95% confidence interval (CI). A positive value of IC0.25 (IC0.25 >0) is the conventional threshold for detecting statistical signals.

In cases where the entire database is not used as a comparator metric, sensitivity analyses are performed, and the reporting odds ratio (ROR) is the preferred measure of disproportionality. The ROR, a frequentist measure of association, is derived from the number of adverse events and the vaccine's contingency table. It is calculated by comparing the probability of the same event occurring with a targeted vaccine to the probability of the same event occurring with all other vaccines in the database. The formula for calculating ROR is as follows: ROR = (a/b) / (c/d), where "a" represents the number of reports for a certain adverse drug reaction, "b" is the number of reports for all other ADRs with a specific drug, "c" is the number of all reports for certain ADRs not related to a specific drug, and "d" is the number of all reports not related to both specific ADRs and drugs. The fact that the 95% lower CI of the ROR is >1.0 is considered an important association between the drug and a certain ADR.

**Supplementary References**

1. Lindquist M, Edwards IR. The WHO Programme for International Drug Monitoring, its database, and the technical support of the Uppsala Monitoring Center. *J Rheumatol* 2001; **28**(5): 1180-7.

2. Lee S, Yang JW, Jung SY, et al. Neuropsychological adverse drug reactions of Remdesivir: analysis using VigiBase, the WHO global database of individual case safety reports. *Eur Rev Med Pharmacol Sci* 2021; **25**(23): 7390-7.

3. Min C. The importance of a World Health Organization international pharmacovigilance database (VigiBase): novel methods for safety monitoring and surveillance of medical products. *Life Cycle* 2022; **2**: e13.

4. Lee K, Lee H, Kwon R, et al. Global burden of vaccine-associated anaphylaxis and their related vaccines, 1967-2023: A comprehensive analysis of the international pharmacovigilance database. *Allergy* 2024; **79**(3): 690-701.

5. Sandberg L, Taavola H, Aoki Y, Chandler R, Norén GN. Risk Factor Considerations in Statistical Signal Detection: Using Subgroup Disproportionality to Uncover Risk Groups for Adverse Drug Reactions in VigiBase. *Drug Saf* 2020; **43**(10): 999-1009.
